# DNA analysis of the last Brazilian unknown soldier’s remains buried in Pistoia (Italy)

**DOI:** 10.1080/20961790.2020.1713453

**Published:** 2020-02-18

**Authors:** Vincenzo Agostini

**Affiliations:** Università Degli Studi Del Piemonte Orientale “A. Avogadro”, Alessandria, Italy

**Keywords:** Forensic sciences, forensic genetics, body remains, bone, tooth, STR polymorphisms, World War II

## Abstract

During World War II, many nations took part in the war. Among the supporters of the Alliance there was also Brazil. In August 1944, under the leadership of President Getúlio Vargas, Brazil declared war on Nazi Germany and took part in the Italian campaign by sending many troops to support the Allies in the Central Italy. Once the conflict was over, the deceased Brazilian soldiers were buried in Pistoia, a few kilometers from Florence. But only in 1960 the Brazilian government authorized the transfer of the dead soldiers to their homeland. Five years later, during the building of the Brazilian Military Votive Monument, still in the Pistoia cemetery, a last body was found but could not be identified: so he was buried as an “unknown soldier”. In December 2012, the Brazilian Embassy in Italy asked for performing forensic genetics analysis for identification purposes on the remains of this last unknown Brazilian soldier. After almost 70 years a complete short tandem repeats (STR) profile was obtained, useful for any relatives searching.

**Key points:**

- Identification of the last Brazilian Unknown Soldier buried in Italy.
- DNA analysis on 70 years old skeletal remains.
- Brazilian soldier’s history during World War II.

## Introduction

### Historical introduction

The ***Força Expedicionária Brasileira*** (in English “Brazilian Expedition Force”), known as *FEB*, was the Brazilian military body that fought with the Allies in Italy, during World War II. Initially constituted of a division of infantry, the armed forces were then joined by three more units of the Brazilian army, among which dreadnoughts and air force. Once the war started, the FEB adopted their famous motto “The Smoking Snake”, referring to a speech of President Getúlio Vargas, during which he said: “It's more likely for a snake to smoke a pipe, than for the FEB to go the front and fight” [1, 2].

In fact, at the beginning of the war, in 1939, Brazil remained neutral, deciding not to fight. This situation changed when, at the beginning of 1942, the US ordered the Brazilian government to give up the island of Fernando de Noronha and the northeastern coast of Brazil for their military bases. In January of the same year, a series of torpedoes were fired against Brazilian merchant ships by Italian–German submarines [1, 2]. The Italian–German attacks had the purpose to intimidate the Brazilian government so that it remained neutral. After several accusatory speeches from Berlin’s radio stations and after several attacks, which caused the death of numerous civilians, the Brazilian government decided to change its status and on 22 August 1942 Brazil declared war to Nazi Germany and to Fascist Italy [1, 2].

However, only after two years, on 2 July 1944, a first unit of the FEB left for Naples under the command of General João Batista Mascarenhas de Morais [3, 4].

The Brazilian soldiers of the FEB became one of the 20 allied divisions at the front and were added to the IV Division of the United States Army, under the command of General Willis D. Crittemberger. The division was part of the 5th American Army guided by General Mark Wayne Clark [5, 6].

The FEB was officially part of the Campaign in Italy and its actions started in the battle in September 1944, in the valley of the Serchio river, north of the town of Lucca. The first victories of the FEB were obtained with numerous conquests. However the FEB had to face the rigid winter between 1944 and 1945, staying on the mountains of the Tuscan-Emilian Apennine, with snow, humidity and temperatures close to −20 °C. Moreover continuous attacks from the enemy tested the physical and psychological resistance of the Brazilian troops already weak and not used to the low temperatures [2].

On 6 June 1945, the Brazilian Ministry of War ordered the FEB units still in Italy be subordinate to the commander of the first military region (1st MR), located in the city of Rio de Janeiro. This meant the dissolution of the contingent [7].

During the war, Brazil lost about 450 soldiers and thirteen officers, in addition to eight pilot-officers of the Brazilian Air Force [7]. Moreover, 2 000 people died because of wounds suffered during the battles and more than 12 000 suffered mutilations or other wounds. In conclusion, of the 25 000 men sent to Italy, more than 22 000 had an active part in the actions [8].

The Brazilian soldiers dead in the action were buried in Pistoia—San Rocco, an important city center near Florence. In 1960, they were transferred into Brazil, in a monument that was built in the Aterro do Flamengo, located in the South area of Rio de Janeiro, to honor the memory of their sacrifice. Five years later, in Pistoia, the construction of the Brazilian Military Monument began [9]. During the work a last body was found but could not be identified: it was decided to leave it in the shrine itself as an “unknown soldier” [4].

In December 2012, the Brazilian Embassy in Italy asked for performing the exhumation of the last Brazilian soldier. The request was to obtain a DNA profile from bone remains in order to look for potential relatives or descendants and perhaps identifying the soldier after about 70 years.

On 6 December 2012, began the exhumation activities of the coffin with the soldier’s remains.

Inside the coffin there was a skeletonized human body.

The body consisted of:

- n. 1 skull + 4 teeth (21, 22, 23, 27); the mandible was not present;
- n. 15 vertebrae + sacral complex;
- n. 23 ribs;
- n. 2 scapulae;
- n. 2 clavicles;
- n. 2 humeri;
- n. 2 radiuses;
- n. 2 ulnae;
- n. 1 acetabulum;
- n. 2 femora;
- n. 2 tibiae;
- n. 2 peronei;
- n. 30 short bones;
- other small bone fragments.

Inside the coffin there were also a glass bottle containing a sheet of paper, fragments of a blanket and some entomological material (most likely puparia) inside the acetabulum.

For the analysis of the short tandem repeat (STR) genetic polymorphisms of human DNA, teeth 21 (upper left central incisor), 22 (upper left lateral incisor) and 23 (upper left canine) were used.

### Molecular introduction

In cases of cadaveric identification, the study of the STR genetic polymorphisms of human DNA must be performed on cadaveric organic remains. In these circumstances, considering also the state of decomposition of the cadaver, it is necessary to look for organic matrices that allow a good DNA extraction and a successful genetic profile typing, which is clear and easy to interpret and to be used in paternity/maternity tests or other identification comparisons [10]. The organic samples that are normally taken during exhumations are nails, bones (usually femura, humeri, or metatarsal/metacarpal bones) and teeth. In skeletal remains, even more especially in teeth, DNA is bond to the inorganic mineral matrix, remaining protected from degradation caused by enzymes and bacteria [11]. In this way, DNA can also be preserved in very old skeletal remains. Usually preservation of DNA is reduced with age. However, that is not always the case as the environments surrounding the skeletal samples have the biggest effect on their preservation [12, 13].

The most significant environmental factors that can alter and damage the DNA structure for forensic purpose are temperature, humidity, pH, the chemical characteristics of the soil and the presence of microorganisms. But temperature and humidity are for sure the most critical points for a good conservation of the DNA [14]. However, often it happens that DNA is well preserved if the organic matrix is preserved in environments/soils rich of salt, with a neutral or slightly alkaline pH, low humidity and low content of humic acid [14].

When it comes to skeletal remains, teeth are the organic matrix of choice for DNA extraction, even after several years: the DNA contained inside the dentin and in the dental pulp is protected by a thick inorganic layer of hydroxyapatite crystals, which create a physical protective barrier, preserving the nucleic acid and slowing down the degradation processes even after several years from the moment of death [15, 16].

For these reasons, in the present work, three dental elements, still in a good morphological and structural state inside the maxilla, were analyzed.

## Materials and methods

### Sample preparation

The soil components and the potential contaminating agents present on the surface of the teeth were physically removed using sterile sandpaper and, subsequently, with ultraviolet (UV) lights and a wash with water and ethanol. Then the teeth were left to dry overnight. The following day they were again exposed to UV light for 30 min per side [17].

For an initial gross fragmentation of the teeth, a scalpel and a mortar were used, previously sterilized in water, bleach and ethanol. For the pulverization, the homogenizer TissueLyser II (Qiagen, Hilden, Germany) and liquid nitrogen were used to obtain fine teeth powder. The sample was treated with metal beads in metal jars, using Grinding Jar Set, Stainless Steel (2 × 10 mL) (Qiagen). The metal jars and teeth fragments were cooled down and then grinded for 2 min at frequency of 30 Hz [17].

At the end of the pulverization step the powder was transferred inside a Falcon tube for the extraction phase.

### DNA extraction

Totally 0.5 g of teeth powder for the DNA extraction process was used, after 24 h of decalcification with 0.5 mol/L EDTA, at a pH of 8.3, following the protocol discussed in Zupanič Pajnič *et al.* [17] in 2010. DNA extraction was performed using organic extraction protocols, with phenol-chloroform and DNA micro kit (Qiagen) [18, 19]. At the same time, a negative extraction control was setted up and gave the expected results.

### Polymerase chain reaction (PCR) analysis

PCR was carried out using the AmpFℓSTR^®^ Identifiler Plus^®^ PCR Amplification Kit (ThermoFisher Scientific, Waltham, MA, USA): 15 autosomal STRs and Amelogenin for gender determination were amplified simultaneously. Negative and positive controls were analyzed and gave the expected results [18, 20].

### DNA typing

Capillary electrophoresis was performed throughout using an ABI PRISM^®^ 310 Genetic Analyzer (ThermoFisher Scientific), with a 50 cm capillary array and POP-4 TM polymer, injecting 1 μL of PCR product, 15 μL of HI-DI TM formamide and 0.35 μL of LIZ500 TM size standard. Software: GeneMapper ID v3.2.1 (ThermoFisher Scientific) [18, 21].

## Results and discussion

After the PCR amplification and sequencing operations, it was possible to type a clear and comprehensible genetic profile, attributable to a male individual and useful for further identification steps.

**Figure 1.**
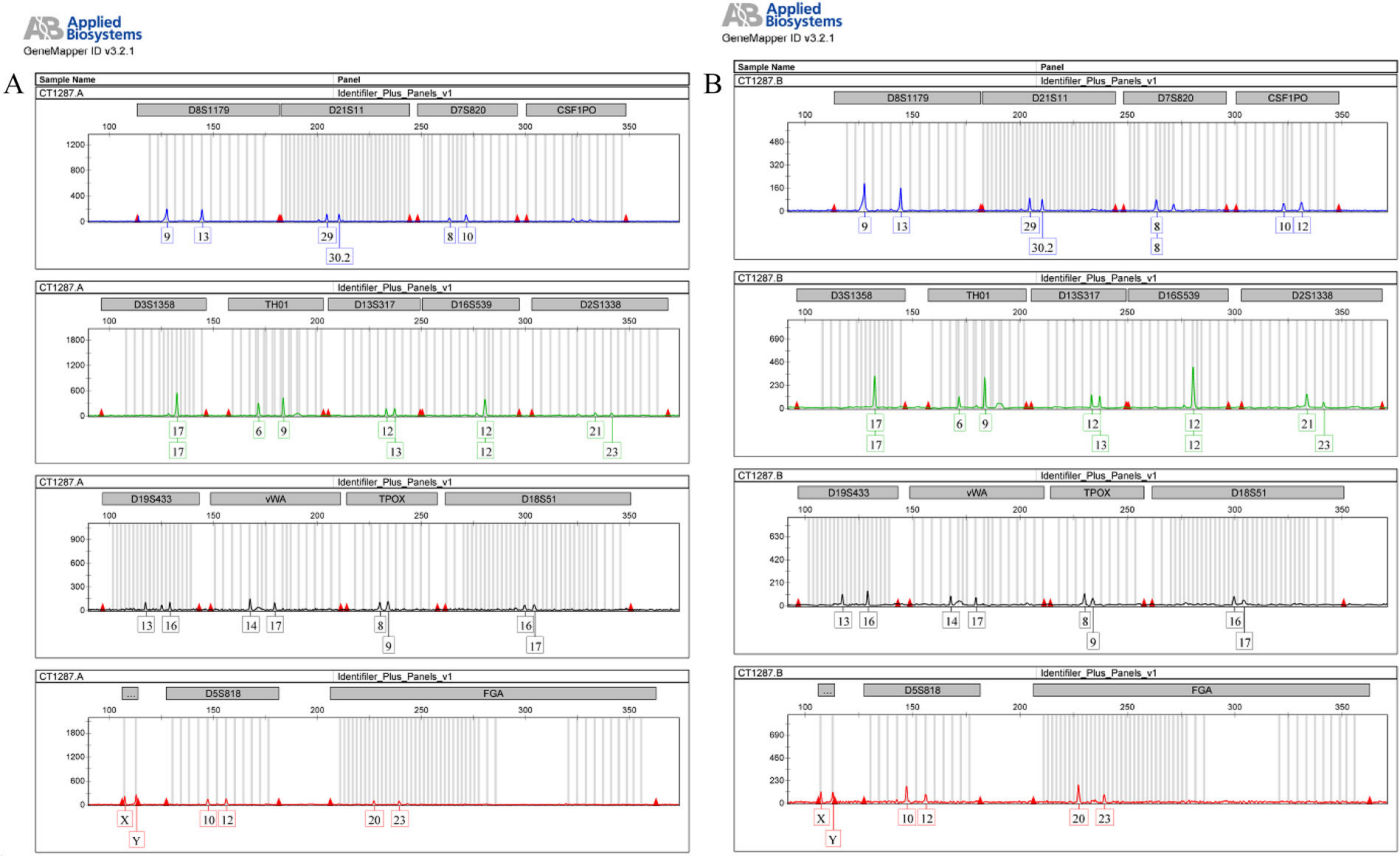
First (A) and second (B) replicate performed on extracted DNA from soldier’s teeth.

Several distinct replicates of the analysis were performed, starting from the extracted DNA, and each of them gave the same result, meaning a repeatable and perfectly superimposable profile, proving the reliability of the obtained data (Figure 1). Negative and positive controls gave the expected results (data not shown).

Genetic data belong surely to the soldier remains, because the teeth were accurately treated and exposed to UV light for long time, in order to eliminate each possible external contamination. Moreover the obtained genetic profile were compared with all the technicians that work in the laboratory in order to exclude any further carryon contamination.

As biostatistical evaluation only the Random Match Probability (RMP) was performed and the evaluation of the genetic profile’s frequency is 11 139 × 10^−21^, in the reference Brazilian population (NIST-STRBase) [22].

Although the skeletal remains found inside the coffin were in a poor state of conservation, in a humid environment and exposed to the action of the microfauna, the dental materials found were sufficient to allow an excellent genetic typing of the subject, even after about 70 years.

## Conclusion

After the exhumation of the last Brazilian Unknown Soldier buried in Pistoia (Italy), only three teeth were collected and analyzed, using human STR polymorphisms analysis. Despite of the poor state conservation, after 70 years, it was possible to obtain a complete, clear and repeatable DNA profile, belonging to a male individual. Such certified profile was sent to the Brazilian Embassy in Italy, in order to submit it to their national DNA database to search for potential relatives and to be able to find at least a descendent and give a name to last Brazilian Unknown Soldier buried in Italy. Even today, however, it has not been possible to find relatives of the soldier and give him a name.

## Author’s contribution

The author was the sole contributor to this paper.

## Compliance with ethical standard

This article does not contain any studies with human participants or animals performed by the author. All procedures performed in studies were in accordance with the ethical standards of the institutional review board of Università del Piemonte Orientale “A. Avogadro”.
